# Impairment of Membrane Lipid Homeostasis by Bichalcone Analog TSWU-BR4 Attenuates Function of GRP78 in Regulation of the Oxidative Balance and Invasion of Cancer Cells

**DOI:** 10.3390/cells9020371

**Published:** 2020-02-05

**Authors:** Tsung-Lin Lee, Shyang-Guang Wang, Wen-Ling Chan, Ching-Hsiao Lee, Tian-Shung Wu, Meng-Liang Lin, Shih-Shun Chen

**Affiliations:** 1 Department of Family Medicine, Chang Bing Show Chwan Memorial Hospital, Changhua 50544, Taiwan; frisbee945@gmail.com; 2 Department of Medical Laboratory Science and Biotechnology, Central Taiwan University of Science and Technology, Taichung 40601, Taiwan; sgwang@ctust.edu.tw; 3 Department of Bioinformatics and Medical Enginerring, Asia University, Taichung, Taiwan; wlchan@asia.edu.tw; 4 Department of Medical Technology, Jen-Teh Junior College of Medicine, Nursing and Management, Miaoli 356, Taiwan; heecs@ms38.hinet.net; 5 Department of Chemistry, National Cheng Kung University, Tainan 70101, Taiwan; tswu@mail.ncku.edu.tw; 6 Department of Medical Laboratory Science and Biotechnology, China Medical University, Taichung 40402, Taiwan

**Keywords:** p85α, ASM, bichalcone analog, ceramide, GRP78, lipid raft, PTEN, ROS

## Abstract

The specialized cholesterol/sphingolipid-rich membrane domains termed lipid rafts are highly dynamic in the cancer cells, which rapidly assemble effector molecules to form a sorting platform essential for oncogenic signaling transduction in response to extra- or intracellular stimuli. Density-based membrane flotation, subcellular fractionation, cell surface biotinylation, and co-immunoprecipitation analyses of bichalcone analog ((*E*)-1-(4-Hydroxy-3-((4-(4-((*E*)-3-(pyridin-3-yl)acryloyl)phenyl)piperazin-1-yl)methyl)phenyl)-3-(pyridin-3-yl)prop-2-en-1-one (TSWU-BR4)-treated cancer cells showed dissociation between GRP78 and p85α conferring the recruitment of PTEN to lipid raft membranes associated with p85α. Ectopic expression of GRP78 could overcome induction of lipid raft membrane-associated p85α–unphosphorylated PTEN complex formation and suppression of GRP78−PI3K−Akt−GTP-Rac1-mediated and GRP78-regulated PERK−Nrf2 antioxidant pathway and cancer cell invasion by TSWU-BR4. Using specific inducer, inhibitor, or short hairpin RNA for ASM demonstrated that induction of the lipid raft membrane localization and activation of ASM by TSWU-BR4 is responsible for perturbing homeostasis of cholesterol and ceramide levels in the lipid raft and ER membranes, leading to alteration of GRP78 membrane trafficking and subsequently inducing p85α–unphosphorylated PTEN complex formation, causing disruption of GRP78−PI3K−Akt−GTP-Rac1-mediated signal and ER membrane-associated GRP78-regulated oxidative stress balance, thus inhibiting cancer cell invasion. The involvement of the enrichment of ceramide to lipid raft membranes in inhibition of NF-κB-mediated MMP-2 expression was confirmed through attenuation of NF-κB activation using C2-ceramide, NF-κB specific inhibitors, ectopic expression of NF-κB p65, MMP-2 promoter-driven luciferase, and NF-κB-dependent reporter genes. In conclusion, localization of ASM in the lipid raft membranes by TSWU-BR4 is a key event for initiating formation of ceramide-enriched lipid raft membrane platforms, which causes delocalization of GRP78 from the lipid raft and ER membranes to the cytosol and formation of p85α–unphosphorylated PTEN complexes to attenuate the GRP78-regulated oxidative stress balance and GRP78−p85α−Akt−GTP-Rac1−NF-κB−MMP-2-mediated cancer cell invasion.

## 1. Introduction

Increased level of cholesterol/sphingolipid-rich lipid rafts in cancer cells is related to high synthesis of lipid and cholesterol by changing in lipid- and cholesterol-associated pathways [1]. The dynamic compartmentalization of the lipid rafts modulates the lateral compartmentalization of receptors at the cell surface to facilitate the interaction of receptor and signaling molecules during advanced and metastatic stages of carcinogenesis [1,2]. The lipid rafts are characterized by low-buoyant density in sucrose gradient centrifugation and by their biochemical property in resistance to extraction with nonionic detergents, such as Triton X-100, NP-40 at 4 °C or Brij 98 at 37 °C [2,3]. The phosphatidylinositol 3-kinase (PI3K)−protein kinase B (Akt) pathway is compartmentalized within the lipid rafts to play functional roles in the regulation of cancer cell metabolism, survival, and invasion [4]. Akt activation is triggered by PI3K induction of generation of phosphatidylinositol-3,4,5-trisphosphate (PIP_3_). PIP_3_ accumulation in the plasma membrane formed by PI3K can also facilitate guanine nucleotide factor-mediated guanosine 5′-triphosphate (GTP) binding to Ras-related C3 botulinum toxin substrate 1 (Rac1) to form GTP-Rac1 [5]. The resulting GTP-Rac1 recruits to the lipid rafts and allows binding of PI3K regulatory subunit p85α and GTP-Rac1 to enhance Akt activation [5]. Importantly, lipid raft recruitment and activation of Akt by PI3K is negatively regulated by the lipid phosphatase activity of phosphatase and tensin homolog deleted from chromosome 10 (PTEN) through dephosphorylating the 3-position of PIP_3_ to phosphatidylinositol-4,5-bisphosphate (PIP_2_) [6,7,8]. The lipid phosphatase activity and membrane association of PTEN is enhanced by modulating the formation of p110-free p85α–unphosphorylated PTEN complexes [9]. Dephosphorization of Ser 380 to Ser 385 cluster of PTEN can impact its enzymatic activity and plasma membrane association [10]. The lipid raft association of PTEN induced by substitution of the cholesterol of the lipid rafts with ceramide was implicated in attenuation of the PI3K−Akt-mediated signal pathway [4,11], indicating that inducing complex formation of p85α with PTEN in the lipid rafts could be a good strategy for blocking PI3K-driven oncogenic activity.

Although the glucose regulated protein 78 (GRP78) is regarded as a major endoplasmic reticulum (ER) chaperon essential for the quality control of protein folding to modulate the ER stress signaling [12], it can form complexes with PI3K on cell surface to increase PI3K−Akt activity and to enhance PIP_3_ generation in cancer cells [13]. The in vitro and in vivo results show that GRP78 is preferentially localized on the cell surface of cancer cells but not in normal cells [14,15,16]. ER is also recognized as a major site of the biosynthesis of cholesterol and lipids [17], as evidenced by involvement of GRP78 in regulating lipid metabolism of cancer cells and its localization in ER [18]. Additionally, GRP78 modulates antioxidant activities of glutathione and NAD(P)H:quinone oxidoreductase by inducing activation of protein kinase RNA-like endoplasmic reticulum kinase and nuclear factor erythroid 2 p45-related factor 2 [19], contributing the protection of cancer cells from oxidative stress-induced damage [20,21]. Increased formation of cell surface GRP78−PI3K complexes in cancer cells confers a beneficial effects on cell survival and resistance to reactive oxygen species (ROS)-induced lipotoxicity [13,22].

Using a Mannich base reaction, a series of novel bichalcones with different substitutions in the B-ring of the chalcone moiety were synthesized, the analog ((*E*)-1-(4-Hydroxy-3-((4-(4-((*E*)-3-(pyridin-3-yl)acryloyl)phenyl)piperazin-1-yl)methyl)phenyl)-3-(pyridin-3-yl)prop-2-en-1-one, or TSWU-BR4) with a pyridyl group at 3-position of the B-ring of the chalcone basic skeleton exhibited the strongest in vitro growth inhibitory activity against human colon adenocarcinoma HT-29 cell line among all TSWU-BR analogs [23]. It was also reported to suppress the generation of nitric oxide (•NO) [23]. •NO produced endogenously by cancer cells acts as a signaling messenger in association with cancer cell growth by modulating oxidative stress [24,25]. Cancer cells combat elevated levels of ROS by increasing NADPH and glutathione levels through an increase in •NO level [25]. The above observations promote us to question whether TSWU-BR4 inhibits cancer cell invasion by deregulating oxidative balance and lipid homeostasis via disturbance of PI3K-regulated signaling. The present study was therefore designed to investigate the underlying mechanisms involved in the inhibition of PI3K signaling pathways by TSWU-BR4 linked to oxidative stress and membrane lipid imbalance sensitivity of cancer cells.

## 2. Materials and Methods

### 2.1. Cell Culture

Epstein–Barr virus-negative human NPC-TW 039 cell line was derived from a 64-year-old male Chinese patient with keratinizing squamous cell carcinoma (WHO type I) [26]. NPC-TW 039 cell line was obtained as previously described [27]. Normal human embryonic lung fibroblasts (WI-38 and MRC-5) and a human pharyngeal squamous carcinoma FaDu cell line were obtained from the Food Industry Research and Development Institute (Hsinchu, Taiwan). The WI-38, MRC-5, and FaDu cell lines were cultured in minimum essential medium (MEM) supplemented with 5% fetal bovine serum (FBS). The NPC-TW 039 cell line was cultured routinely in Dulbecco’s Modified Eagle’s Medium (DMEM) supplemented with 5% FBS. These cell lines were grown in 10 cm tissue culture dishes at 37 °C in a humidified incubator containing 5% CO_2_.

### 2.2. Chemicals, Reagents, and Plasmids

Actinomycin D (ActD), N-acetyl-D-sphingosine (C2-ceramide), amitriptyline, Brij 98, crystal violet, 1,2-dioleoyl-*sn*-glycero-3-phosphocholine (DOPC), glutathione agarose beads, imipramine, propidium iodide (PI), pyrrolidine dithiocarbamate (PDTC), Tris-HCl, Triton X-100, 3-(4,5-dimethylthiazol-2-yl)-2,5-diphenyltetrazolium bromide (MTT), and N-*p*-tosyl-_L_-phenyl-alanine chloromethyl ketone (TPCK) were obtained from Sigma-Aldrich (St. Louis, MO, USA). TSWU-BR4 was prepared as previously described [23]. The purity of TSWU-BR4 by HPLC analysis was 96.68%. DMSO and potassium phosphate were purchased from Merck (Darmstadt, Germany). Lipofectamine 2000 was obtained from Invitrogen (Carlsbad, CA, USA). DMEM, FBS, penicillin-streptomycin, trypsin-EDTA, and glutamine were obtained from Gibco BRL (Grand Island, NY, USA). The Annexin V-FITC Apoptosis Detection Kit I was obtained from BD PharMingen (San Diego, CA, USA). Engelbreth-Holm-Swarm sarcoma tumor (EHS) extract Matrigel was obtained from BD Biosciences (Bedford, MA, USA). The caspase-3 activity assay kit was purchased from OncoImmunin (Gaithersburg, MD, USA). The caspase-3 inhibitor Ac-DEVD-CMK and N-acetyl-L-cysteine (NAC) were purchased from Calbiochem (San Diego, CA, USA). Centricon YM-100 was obtained from Millipore. Acid sphingomyelinase (ASM) Assay Kit (Fluorimetric) was obtained from Abcam (Cambridge, MA, USA). The Amplex Red cholesterol assay kit was purchased from Molecular Probe (Eugene, OR, USA). The MMP-2 and MMP-9 promoter luciferase reporter constructs were prepared as described previously [28]. The β-galactosidase expression vector and pCH110 were purchased from Amersham Pharmacia Biotech (Piscataway, NJ, USA). pGFP shRNA, pGRP78, and pCMV4-NF-κB p65 were obtained from Addgene (Cambridge, MA, USA). The luciferase assay kit was obtained from Promega (Madison, MI, USA). ASM shRNA phasmid and Western blot luminol reagent were purchased from Santa Cruz Biotechnology.

### 2.3. Antibodies

Antibodies against MMP-1 and MMP-9 were purchased from Oncogene Research Product (Cambridge, MA, USA). Antibody against caspase-3 was obtained from Calbiochem (San Diego, CA, USA). Anti-Akt, -phospho (p)-Akt (Ser 473), and -NF-κB p65 antibodies were purchased from BD PharMingen. Anti-p-NF-κB (Ser 536) antibody was obtained from Cell Signaling Technologies (Boston, MA, USA). Antibodies directed against ASM and calnexin were obtained from Abcam (Cambridge, MA, USA). Antibodies against CD55, CD71, GRP78, nucleolin, p110α, poly (ADP-ribose) polymerase (PARP), and Rac1 were purchased from Santa Cruz Biotechnology. Anti-pan-cadherin, anti-PTEN, anti-p-PTEN (Ser 380), and anti-p-PTEN (Ser 380/Thr 382/Ser 385) antibodies were obtained from Thermo Fisher Scientific (New York, NY, USA). Antibody against β-actin was obtained from Sigma-Aldrich. Peroxidase-conjugated anti-mouse, -goat, and -rabbit IgG secondary antibodies were purchased from Jackson ImmunoResearch Laboratory (West Grove, PA, USA).

### 2.4. Cell Viability Assay

Cell viability was assessed by fluorescence-activated cell sorting (FACS) analysis of cellular PI uptake [28]. Cells were seeded at 3 × 10^4^ cells/well in 24-well tissue culture plates. The cells were grown overnight to ~60% confluence and treated with either DMSO as the vehicle control or TSWU-BR4 for 36 h. Serving as a vehicle control, DMSO was diluted in culture medium to the same final concentration of DMSO (0.1%; vol/vol) as TSWU-BR4. At the end of the incubation, the treated cells were harvested and stained with PI solution (10 μg/mL) in phosphate-buffered saline (PBS). The samples were analyzed on a FACSCount flow cytometer (BD Biosciences, Franklin Lakes, NJ, USA). Cell Quest software (BD Biosciences, Franklin Lakes, NJ, USA) was used to analyze the results. PI-negative populations were defined as viable cells

### 2.5. Assays for the Detection of Caspase-3 Activity and Early Apoptotic Cells

Caspase-3 activity was measured using the PhiPhiLux G1D2 kit (OncoImmunin, College Park, MD, USA) according to the manufacturer’s protocols. Briefly, treated cells were incubated with PhiPhiLux fluorogenic Caspase substrate at 37 °C for 1 h and then analyzed using a FACSCount flow cytometer. For the Annexin-V binding assays, treated cells were harvested and stained with fluorescein isothiocyanate-labeled Annexin-V and PI according to the manufacturer’s protocols. The samples were assessed using a FACSCount flow cytometer, and the results were analyzed using CellQuest software.

### 2.6. Density-Based Membrane *Flotation Technique*

Treated cells were washed twice in ice-cold PBS before being removed from dishes by scraping. Cells were then harvested by centrifugation, resuspended in 1 mL of hypotonic lysis buffer (10 mM Tris (pH 7.5), 10 mM KCl, 5 mM MgCl_2_) containing 0.5% Brij 98, incubated at 37 °C for 5 min, and ruptured by 20 passages through a 25-gauge hypodermic needle. Unbroken cells and nuclei were removed by centrifugation at 1000× *g* for 5 min in a microcentrifuge at 4 °C. The crude homogenates were maintained on ice for an additional 5 min, mixed with 3 mL of 72% sucrose, and overlaid with 4 mL of 55% sucrose and 1.5 mL of 10% sucrose; all of the sucrose solutions were dissolved in low-salt buffer (50 mM Tris-HCl (pH 7.5), 25 mM KCl, 5 mM MgCl_2_). The samples were centrifuged for 14 h in a Beckman SW41 rotor at 38,000 rpm and 4 °C. Fractions were collected from the top of the gradient in 1 mL increments and concentrated to approximately 100 μL by passage through a 50 kDa Centricon filter.

### 2.7. Isolation of ER and Cytosolic Fractions

Fractionations of ER and cytosolic membranes were performed according to the protocol of Zong et al. [29]. The treated cells were washed twice with ice-cold PBS and scraped into a 200 mM sucrose solution containing 25 mM HEPES (pH 7.5), 10 mM KCl, 15 mM MgCl_2_, 1 mM EDTA, 1 mM EGTA, and 1 μg/mL aprotinin. The cells were disrupted by passage through a 26-gauge hypodermic needle 30 times and then centrifuged for 10 min in an Eppendorf microcentrifuge (5804R) at 750× *g* at 4 °C to remove unlysed cells and nuclei. The supernatant was collected and then centrifuged for 20 min at 10,000× *g* at 4 °C to form a new supernatant and pellet. The resulting supernatant was further centrifuged at 100,000× *g* for 1 h at 4 °C. The new supernatant was saved as the cytosolic (C) fraction, and the pellet was reserved as the ER fraction. The resulting ER and C fractions were lysed in RIPA buffer (1% sodium deoxycholate, 0.1% SDS, 1% Triton X-100, 10 mM Tris-HCl (pH 8.0), and 0.14 M NaCl) for Western blot analysis. The purity of each subcellular fraction was confirmed by Western blotting using specific antibodies against the ER marker calnexin and the cytosol marker α-tubulin.

### 2.8. Subcellular Fractionation

Subcellular fractionation was performed according to the protocol reported by Taha et al. [30]. The treated cells were washed twice with ice-cold PBS and scraped into a detergent-free lysis buffer (10 mM Tris/HCl (pH 7.4), 10 mM NaCl, 0.5 mM MgCl_2_, and EDTA-free protease inhibitor cocktail). The suspension of cells was homogenized using a prechilled 7 mL Dounce homogenizer and then centrifuged at 1200× *g* for 5 min at 4 °C. The pellet was resuspended in 250 mM sucrose solution containing 10 mM MgCl_2_ and centrifuged through an 880 mM sucrose cushion containing 0.5 mM MgCl_2_ at 1200× *g* for 10 min. The resulting supernatant and pellet served as cytosolic and crude nuclear fractions, respectively. The supernatant was collected and then centrifuged for 5 min at 1200× *g* and 4 °C. The resulting new supernatant was further subjected to a 16,000× *g* centrifugation step for 10 min at 4 °C to isolate the heavy membrane pellet. The heavy membrane pellet was reserved as the plasma membrane fraction and lysed in RIPA buffer (1% sodium deoxycholate, 0.1% SDS, 1% Triton X-100, 10 mM Tris-HCl (pH 8.0), and 0.14 M NaCl) for Western blot analysis of the coimmunoprecipitation experiment. The purity of each subcellular fraction was confirmed by Western blotting using a specific antibody against the nuclear marker nucleolin, the cytosolic marker α-tubulin, or the plasma membrane marker cadherin.

### 2.9. Western Blot and Co-Immunoprecipitation

Treated or transfected cells were lysed and subjected to Western blotting as described previously [31]. For the co-immunoprecipitation assays, cellular extracts were immunoprecipitated with anti-p85α, anti-RP78 antibodies, or with normal control IgG, and then incubated with protein A agarose beads as previously described [31]. After incubation at 4 °C for 2 h, the immune complexes were analyzed by 10% SDS-PAGE and immunoblotting with anti-GRP78, anti-p85α, anti-110α, anti-Rac1, anti-p-Akt (Ser 473), and anti-Akt antibodies. Densitometric measurements of the band in Western blot analysis were performed using computing densitometer and ImageQuant software (Molecular Dynamics, Sunnyvale, CA, USA).

### 2.10. Cell Surface Biotinylation

This assay was performed as previously described [28,32]. Briefly, treated cells were washed twice in ice-cold PBS and incubated with 0.5 mg/mL of EZ-Link Sulfo-NHS-SS-Biotin (Pierce, Rockford, IL, USA) for 30 min at 4 °C. Biotinylated cells were washed twice in ice-cold PBS and treated with 50 mM NH_4_Cl for 10 min at 4 °C to stop the biotinylation reaction. Avidin-agarose beads (Pierce, Rockford, IL, USA) were then added to the biotinylated cells, and the mixture was incubated with gentle rocking at 4 °C for 16 h. The beads were pelleted and washed three times with 500 μL of ice-cold PBS. Bound proteins were mixed with 1× SDS sample buffer and incubated for 5 min at 100 °C. The proteins were then separated by 10% SDS-PAGE and immunoblotted with antibody against GRP78.

### 2.11. Measurement of Cell Surface or Intracellular GRP78 by Flow Cytometry

This assay was performed as previously described [32]. Briefly, treated cells (1 × 10^6^) were detached from culture plates by 1 mM EDTA, washed twice with PBS, and incubated with 10% normal human serum in PBS for 20 min on ice to block fragment crystallizable receptors (FcRs) on the cell surface. The cells were washed three times with ice-cold PBS and then incubated with 0.5 μg anti-GRP78 for 30 min on ice in 50 μL of staining buffer (2% FCS in 1× PBS). For the staining of intracellular GRP78, treated cells (1 × 10^6^) were formaldehyde fixed and permeabilized with 0.03% saponin. Intracellular staining was performed in 0.03% saponin in 1× PBS with anti-GRP78. After washing with staining buffer, cells were incubated with fluorescein isothiocyanate (FITC)-conjugated secondary antibody and analyzed on a FACSCount flow cytometer [33].

### 2.12. Rac1 Activation Assay

Treated cells or caveolin-1 and CD55-riched DRM fractions prepared from treated cells were lysed by incubation with Rac1 lysis buffer (50 mM Tris-HCl (pH 7.4), 100 mM NaCl, 1 mM MgCl_2_, 20 mM β-glycerophosphate (pH 7.5), 1% NP-40, 10% glycerol, 10 mM NaF, 2 mM Na_3_VO_4_, 5 mM dithiothreitol, 0.5 mM phenylmethylsulfonyl fluoride, 1 μg/mL leupeptin, and 1 μg/mL pepstatin) for 15 min at 4 °C. The lysates were centrifuged at 14,000× *g* for 10 min in a microcentrifuge at 4 °C. The lysates and immunoprecipitated complexes were incubated with 40 μg of bacterially expressed glutathione-S-transferase (GST)-PAK-CD fusion protein prebound to glutathione agarose beads for 30 min at 4 °C. The beads were pelleted, washed with 500 μL of Rac1 lysis buffer, mixed with 1× SDS sample buffer (50 mM Tris-HCl (pH 6.8), 2% SDS, 0.1% bromophenol blue, 10% glycerol, and 100 mM dithiothreitol) and incubated for 5 min at 100 °C. The samples were then separated by 10% SDS-PAGE and immunoblotted with an antibody against Rac1.

### 2.13. Determination of Cholesterol, Sphingomyelin, and Ceramide

Fifty microliter of cell lysates, caveolin-1 and CD55-riched DRM fractions, or ER fractions were extracted with 200 μL chloroform plus 200 μL methanol and then subjected to centrifugation at 12,000 rpm for 5 min. The bottom layer was collected and then evaporated under vacuum to a small pellet. The pellet was dissolved in 50 μL ethanol. Cholesterol levels were determined using an Amplex Red Cholesterol Assay Kit according to the manufacturer’s protocol. The amount of *s*phingomyelin and ceramide was quantified by thin-layer chromatography as described by Dobrowsky et al. [34].

### 2.14. Measurement ofMMP-2Promoter Activity

Both assays were performed as previously described [35]. Briefly, the pGL3-basic (vector), pGL3-control (control), or MMP-2 promoter promoter plasmids were co-transfected with a β-galactosidase expression vector (pCH110) (10:1) into cells using Lipofectamine 2000 following the manufacturer’s protocols (Invitrogen Life Technology, Carlsbad, CA, USA). At 12 h post transfection, the cells were treated with vehicle, TSWU-BR4, or inhibitor for an additional 36 h. For the overexpression assays, an MMP-2 promoter plasmid and a plasmid expressing NF-κB p65 were transfected into the cells using Lipofectamine 2000. The β-galactosidase expression vector pCH110 was included as an internal control. After 12 h of transfection, the cells were treated with vehicle, TSWU-BR4, or C2-ceramide for an additional 36 h. The cell lysates were harvested, and the protein expression and luciferase activities were assessed as previously described [35].

### 2.15. In Vitro Invasion Assay

The in vitro invasion assay was performed in triplicate as previously described [35] using transwell chamber units with 8 μm pore polycarbonate membranes. Briefly, approximately 1 × 10^4^ cells were resuspended in 200 μL of serum-free DMEM, seeded into the upper chambers of the Matrigel-coated transwell plates and treated with vehicle or TSWU-BR4. Three hundred microliters of the same medium containing 5% FBS was placed in the lower chamber. After 24 or 48 h incubation periods, the cells on the upper surface of the transwell membranes were removed by wiping with a cotton swab, and the cells that had migrated to the lower side of the membrane were stained with a 2% crystal violet solution. For each treatment condition, 10 randomized fields were counted using a light microscope at ×200 magnification. The number of invading cells in each experiment was normalized using the MTT assay (see below) to correct for the effects on cellular proliferation of treatment with vehicle or TSWU-BR4 (normalized invading cell number = counted invading cell number/value of OD_570_).

### 2.16. Plasmid Transfection

Cells (at 70% confluency in a 12-well plate) were transfected with GRP78, ASM shRNA, GRP shRNA, and NF-κB p65 expression plasmids using Lipofectamine 2000. The expression of GRP78, ASM, and NF-κB p65 in the transfected cells was assessed by Western blot using specific antibodies against GRP78, ASM, or NF-κB p65.

### 2.17. NF-κB Promoter Activity

The assay was performed as previously described [35]. Briefly, pTAI-SEAP, pNF-κB-SEAP, or pSEAP2 plasmids were co-transfected into cells with an EGFP expression vector (at a 10:1 ratio). At 8 h post-transfection, the cells were incubated with medium containing vehicle (-), TSWU-BR4 (300 nM), C2-ceramide (6 μM), TPCK (10 μM), or PDTC (10 μM) for the indicated periods. For the overexpression assay, overnight cultured cells were co-transfected with the pNF-κB-SEAP and EGFP expression vectors and an NF-κB p65 expression plasmid (10:10:1) for 24 h. The SEAP activity in the medium was evaluated as previously described [35].

### 2.18. Statistical Analysis

Statistical calculations of the data were performed using the unpaired Student’s *t*-test and one-way ANOVA. *p* < 0.05 was considered statistically significant.

## 3. Results

### 3.1. Delocalization of Cell Surface GRP78 by TSWU-BR4 Induces the Lipid Raft Membrane Localization of Unphosphorylated PTEN to Affect Cancer Cell Invasion

**Figure 1:**
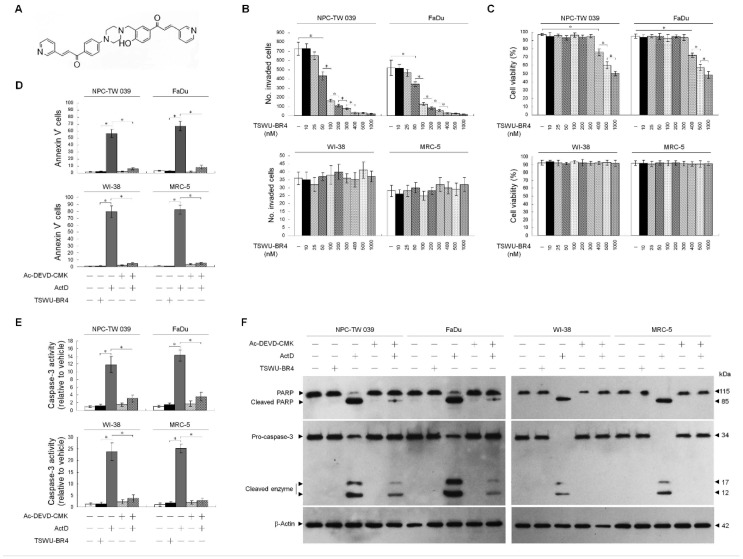
((*E*)-1-(4-Hydroxy-3-((4-(4-((*E*)-3-(pyridin-3-yl)acryloyl)phenyl)piperazin-1-yl)methyl)phenyl)-3-(pyridin-3-yl)prop-2-en-1-one (TSWU-BR4) inhibits the invasive activity of cancer cells. (**A**) The structure of TSWU-BR4. (**B**,**C**) The effect of TSWU- BR4 on cancer cell invasion. Cells were treated with vehicle (−) or the indicated concentrations of TSWU-BR4 for 36 h. The number of invaded cells was assessed using the Matrigel invasion assay. Cell viability was determined by the flow cytometric analysis of PI uptake. The values are presented as the means ± standard error (S.E.M.) of three independent experiments. * *p* < 0.05: significantly different from vehicle (−) or TSWU-BR4-treated cells. (**D**–**F**) The effect of TSWU-BR4 on the induction of cancer cell apoptosis. After a 36 h treatment with vehicle, TSWU-BR4 (300 nM), ActD (10 μM), or ActD and Ac-DEVD-CMK (8 μM), the percentage of Annexin V+ cells and caspase-3 activity were determined using flow cytometry. The values are presented as the S.E.M. of three independent experiments. * *p* < 0.05: significantly different from TSWU-BR4− or ActD-treated cells. The levels of PARP and caspase-3 in the cell lysates were determined by Western blot analysis with specific antibodies. β-Actin was used as an internal control for sample loading.

While investigating the cytotoxic action of the bichalcone analog TSWU-BR4 (Figure 1A) on human cancer cell lines, we found that TSWU-BR4 exhibits a strong inhibitory effect on the growth of both nasopharyngeal carcinoma NPC-TW039 and pharyngeal squamous carcinoma FaDu cells. We were therefore to determine whether treatment with TSWU-BR4 could decrease invasion activity of the most sensitive NPC-TW 039 and FaDu cells using a Matrigel invasion assay. TSWU-BR4 showed a dose-dependent effect, with 300 nM being significantly more effective at inhibiting cancer cell invasion than the vehicle (Figure 1B). A flow cytometric analysis of PI uptake showed that 300 nM TSWU-BR4 did not affect cancer cell viability (Figure 1C). In contrast, the invasion and viability of normal human WI-38 and MRC-5 fibroblasts were not affected by 300 nM TSWU-BR4 (Figure 1B,C). To investigate whether the reduction in cancer cell invasion was due to apoptosis triggered by TSWU-BR4, apoptotic cells and caspase-3 activity were quantified by flow cytometry and determined by Western blot. Compared with the vehicle-treated cells, treatment with 300 nM TSWU-BR4 did not induce apoptosis as indicated by Annexin V-binding, caspase-3 activity, and the cleavage of caspase-3 and poly (ADP-ribose) polymerase (PARP). Exposure of cancer cells or normal human fibroblasts to the transcriptional inhibitor actinomycin D (ActD) resulted in increases in the percentage of AnnexinV^+^ cells and activate caspase-3 and caused proteolytic cleavage of inactive pro-caspase-3 and PARP form into activated cleaving enzymes, whereas this process was suppressed by the caspase-3 inhibitor Ac-DEVD-CMK (Figure 1D–F). Thus, a concentration of 300 nM was used to treat cancer cells in all subsequent experiments. These results indicate that non-apoptotic concentration of TSWU-BR4 selectively inhibited the invasion of cancer cells.

**Figure 2:**
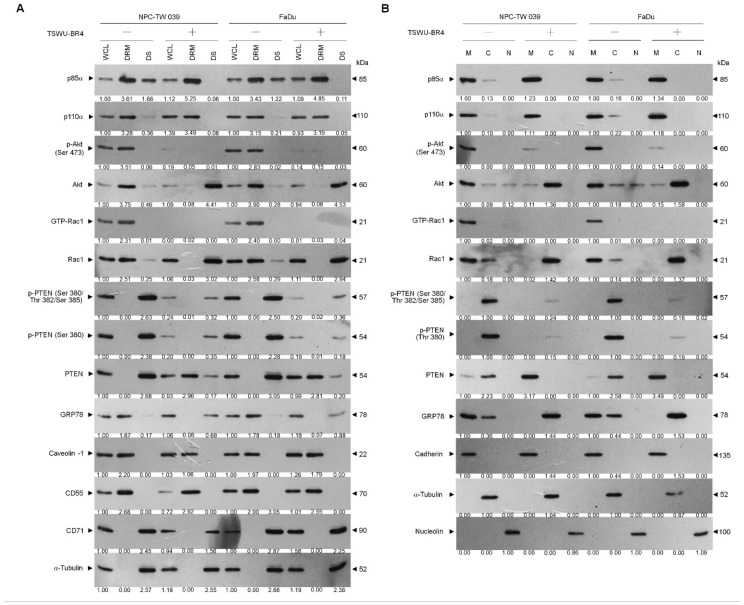
TSWU-BR4 changes the lipid raft membrane localization of GRP78 and PTEN. (**A**,**B**) Cells were treated with vehicle (−) or TSWU-BR4 (300 nM) for 36 h. Detergent-resistant membrane (DRM) and detergent-soluble (DS) fractions were prepared by flotation on a sucrose density gradient. Subcellular plasma membrane (M), cytosolic (C), and nuclear (N) fractions were separated by differential centrifugation. The levels of the indicated proteins in the lysates of vehicle- or TSWU-BR4-treated DRM, DS, M, C, and N fractions were determined by Western blot analysis using specific antibodies. Antibodies against cadherin, α-tubulin, and nucleolin were used as internal controls for the plasma membrane, cytosol, and nucleus, respectively. β-Actin was used as an internal control for sample loading.

Suppressed phosphorylation of Akt (Ser 473) and PTEN (Ser 380/Thr 382/Ser 385) as well as activation of Rac1 were observed in the whole cell lysates (WCL) of TSWU-BR4-treated cells (Figure 2A). To investigate whether inhibition of cancer cell invasion by TSWU-BR4 results in a dysregulation of the lipid raft compartmentalization of phosphatidylinositol 3-kinase (PI3K)−protein kinase B (Akt) signaling-regulated molecules, a sucrose density-based membrane flotation experiments after 1% Brij 98 solubilization of TSWU-BR4-treated cells was performed to isolate detergent-resistant membranes (DRMs), which are biochemically defined as the lipid rafts [2]. Western blot analysis showed that p85α, p110α, Ser 473 phosphorylated Akt, active Rac1 (GTP-bound Rac1; GTP-Rac1), and GRP78 were mainly present in the DRM fractions of vehicle-treated cells and colocalized with the lipid raft markers caveolin-1 and CD55 but not with the non-lipid raft marker CD71, whereas small amounts of p85α, p110α, unphosphorylated Akt, inactive Rac1, and GRP78 were confined in the detergent-soluble (DS) fractions. The phosphatase-inactive form of phospho-PTEN (p-PTEN) (Ser 380/Thr 382/Ser 385) was only detected in the DS fractions of vehicle-treated cells, while the biologically active form of PTEN (unphosphorylated PTEN; PTEN) was not found in the DRM fractions. Cells treated with TSWU-BR4 showed enhanced localization of p85α to the lipid rafts, for which was observed that p85α was increased in the DRM fractions of both cancer cells with a concomitant decrease in the DS fractions. The level of unphosphorylated PTEN protein in the DRM fractions was increased after TSWU-BR4 treatment, correlated with an accompanying decrease in the p-PTEN (Ser 380/Thr 382/Ser 385) level of DS fractions. However, TSWU-BR4 treatment causes decreased levels of p-Akt, GTP-Rac1, and GRP78 in the DRM fractions by increasing the amount of unphosphorylated Akt, inactive Rac1, and GRP78 in the DS fractions (Figure 2A). To verify the membrane localization of PI3K−Akt pathway-related molecules, TSWU-BR4-treated cells were subjected to subcellular fractionation. Under vehicle treatment conditions, p85α, p110α, p-Akt (Ser 473), GTP-Rac1, and GRP78 were mainly found in the membrane (M) fractions and associated with the plasma membrane marker cadherin [36], with small amounts of p85α, p110α, unphosphorylated Akt, inactive Rac1, and GRP78 observed in the cytosolic (C) fractions. In addition to the membrane localization of little unphosphorylated PTEN, we found that phosphorylated PTEN (Ser 380/Thr 382/Ser 385) was highly concentrated in the cytosol. TSWU-BR4 treatment caused an elevated p85α level in the M fractions by decreasing the level of proteins in the C fractions. Treatment of TSWU-BR4 also led to an enhanced localization of unphosphorylated PTEN to the M fractions. Attenuated activation of Akt and Rac1 by TSWU-BR4 correlated with the absence of p-Akt (Ser 473) and GTP-Rac1 in the M fractions and increase levels of unphosphorylated Akt and inactive Rac1 in the C fractions. GRP78 was not present in the M fractions; instead it was localized abundantly to the C fractions of TSWU-BR4-treated cells (Figure 2B). These results suggest that TSWU-BR4 enhances localization of unphosphorylated PTEN to the lipid raft membranes by suppressing the lipid raft membrane association and activation of Akt, Rac1, and GRP78.

### 3.2. Recruitment of Unphosphorylated PTEN to the Lipid Raft Membranes to Form a Complex with p85α in TSWU-BR4-Treated Cells

**Figure 3:**
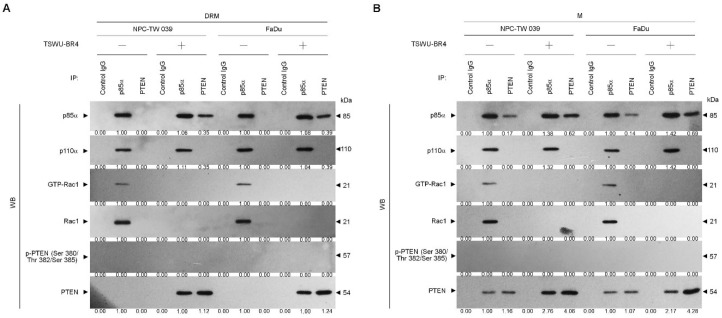
TSWU-BR4 induces p85α–unphosphorylated PTEN complex formation (**A**,**B**) Coimmunoprecipitation of p85α, p110α, Rac1, and PTEN was performed using the DRM and M fractions prepared from the cells treated as described above. The antibody used for coimmunoprecipitation is indicated at the top. The proteins from the immunoprecipitated complexes were detected using Western blotting with specific antibodies. Normal IgG was used as a control for antibody specificity.

As the plasma membrane localization and negative regulator activity controlling the Akt activation of PTNE are modulated by p85α through the formation of p110α-free p85α-unphosphorylated PTEN complexes in the plasma membrane [9], we next examined whether TSWU-BR4 treatment affected the physical interaction between p85α, p110α, Rac1, or PTEN in the lipid raft membranes. In vehicle-treated cells, p110α and GTP-Rac1 were coimmunoprecipitated from the DRM fractions using an antibody specific for p85α; however, immunoprecipitated p85α formed complexes with p110α, GTP-Rac1, and PTEN in the M fractions. Immunoprecipitation of PTEN, followed by immunoblotting for p85α, p110α, p-PTEN (Ser 380/Thr 382/Ser 385), PTEN, and GTP-Rac1 revealed a small amounts of p85α−unphosphorylated PTEN complexes in the M fractions but not in the DRM fractions, indicating that p85α formed a complex with p110α and GTP-Rac1 but did not interact with unphosphorylated PTEN in the lipid raft membranes of vehicle-treated cells. When the cells were subjected to TSWU-BR4 exposure, anti-p85α immunoprecipitations from the DRM and M fractions contained p85α:p110α:unphosphorylated PTEN. Reprecipitation with anti-PTEN antibodies, followed by anti-p85α, -p110α, -Rac1, PTEN, and -p-PTEN (Ser 380/Thr 382/Ser 385) immunoblot analysis was carried out to confirm that a complex with p85α and unphosphorylated PTEN was detected in the DRM and M fractions of TSWU-BR4-treated cells (Figure 3A,B). These observations indicate that TSWU-BR4 induces an association between p85α and unphosphorylated PTEN in the lipid raft membranes of cancer cells.

### 3.3. TSWU-BR4-Induced Dissociation of GRP78 and p85α Leads to the Complex Formation of Lipid Raft Membrane-Associated p85α–Unphosphorylated PTEN and Subsequently Causes Increased ROS Accumulation and Decreased Levels of Cholesterol and Invaded Cells

**Figure 4:**
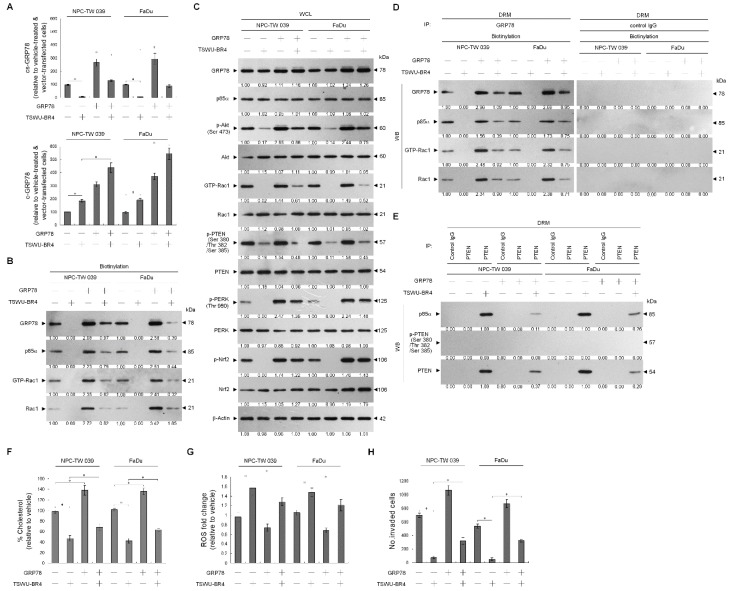
Ectopic expression of GRP78 overcomes TSWU-BR4-induced formation of p85α–unphosphorylated PTEN and inhibition of GRP78-mediated oxidative stress balance and cancer cell invasion. At 12 h after transfection with the GRP78-expressing plasmids or empty vector control, the cells were treated with TSWU-BR4 (300 nM) or TSWU-BR4 (300 nM) plus N-acetyl-L-cysteine (NAC) (100 μM) for an additional 36 h. (**A**) The GRP78 cell surface (cs) and cytosolic (c) levels were determined by flow cytometry. (**B**) Biotinylated proteins were pulled down using streptavidin agarose beads. The biotin-streptavidin complexes were immunoblotted with GRP78, p85α, and Rac1 antibodies. (**C**) The levels of the indicated proteins in the lysates of vehicle- or TSWU-BR4-treated GRP78 transfected cells were determined by Western blot analysis using specific antibodies. β-Actin was used as an internal control for sample loading. The values above the figures represent relative density of the bands normalized to β-actin. (**D**) The GRP78 used for co-immunoprecipitation is indicated at the top of the figure. The biotin-streptavidin complexes in the lipid rafts were immunoblotted with indicated antibodies. (**E**) The antibodies used for co-immunoprecipitation are indicated at the top of the figure. The proteins in the immunoprecipitated complexes from the DRM were analyzed by Western blot using specific antibodies. (**F**) The total cholesterol levels in the whole lysates of the treated cells were measured using the Amplex Red Cholesterol Assay Kit. (**G**) The generation of ROS was monitored by measuring increased fluorescence of 2,7-dichlorodihydrofluorescein by flow cytometry. (**H**) The invaded cell numbers were assessed by the Matrigel invasion assays. The values are presented as the S.E.M. of three independent experiments. *Significantly different at *p* < 0.05.

Given the roles of GRP78 in regulating PI3K−Akt activity and endoplasmic reticulum (ER) oxidative stress [13,21], we next examined whether induced targeting of PTEN with p85α in the lipid raft membranes by TSWU-BR4 was associated with delocalization of GRP78 from the membrane to the cytosol. Flow cytometric analysis showed that the cell surface localization of GRP78 was suppressed, which was associated with an increased level of cytosolic GRP78 by TSWU-BR4 (Figure 4A and Supplementary Figure S1). Elution of streptavidin-agarose bead from the biotinylated protein extracts of vehicle-treated control vector-transfected cells, immunoblotting with anti-GRP78, -p85α, and -Rac1 antibodies detected GRP78−p85α−GTP-Rac1 complexes. The presence of TSWU-BR4 resulted in loss of biotinylated GRP78−p85α−GTP-Rac1 complexes (Figure 4B). TSWU-BR4 prevented the activation of GRP78-regulated protein kinase/RNA-like endoplasmic reticulum kinase/(PERK)−nuclear factor erythroid-2-related factor 2 (Nrf2) antioxidant pathway as illustrated by suppressing phosphorylation of PERK (Thr 980) and Nrf2 and causing an increased production of ROS (Figure 4C,G). Treatment with TSWU-BR4 also reduced cellular cholesterol content by 56% (Figure 4F). In vehicle-treated cells, overexpression of GRP78 resulted in elevated levels of cell surface and intracellular GRP78 that simultaneously increased levels of p-Akt (Ser 473), GTP-Rac1, p-PERK (Thr 980), p-Nrf2, cholesterol, and invaded cells and decreased ROS level (Figure 4A,C,F,G,H). Western blot analysis of streptavidin-agarose bead-bound protein from biotinylated cells further confirmed an increased detection of the cell surface-associated GRP78, p85α, and GTP-Rac1 on the vehicle-treated ectopic GRP78-expressing cells (Figure 4B). Co-immunoprecipitation of cell surface-biotinylated proteins from the DRM fractions isolated with streptavidin-agarose beads using antibody specific for GRP78 revealed that an increase in the recruitment of GRP78−p85α−GTP-Rac1 complexes was detected in the lipid raft membranes of vehicle-treated GRP78-transfected cells, when compared with vehicle-treated control vector-transfected cells (Figure 4D). Forced cell surface and lipid raft membrane targeting of GRP78 by ectopic expression should in turn overcome the induced effect of TSWU-BR4 on the suppression of lipid raft membrane-associated GRP78−p85α−GTP-Rac1 complex formation, Akt (Ser 473) phosphorylation, PERK (Thr 980) phosphorylation, Nrf2 phosphorylation, cholesterol, and cell invasion (Figure 4B,D,F,H). Overexpression of GRP78 also decreased the induction of the formation of lipid raft membrane-associated p85α–unphosphorylated PTEN complexes and ROS level in the presence of TSWU-BR4 (Figure 4E,G). ROS scavenger N-acetyl-L-cysteine (NAC) co-treatment suppressed the increase of ROS; however, NAC can only partially overcome from TSWU-BR4-induced decrease of cholesterol and invaded cell levels. These results indicate that TSWU-BR4 attenuates the association of GRP78 with p85α at the lipid raft membranes to induce the formation of lipid raft membrane-associated p85α–unphosphorylated PTEN complexes, thereby suppressing the regulatory effects of GRP78 on the ER stress-mediated cholesterol, ROS generation, and cell invasion.

### 3.4. Activation of ASM by TSWU-BR4 Deregulates Membrane Trafficking of GRP78, Causes the Lipid Raft Membrane-Associated p85α–Unphosphorylated PTEN Complex Formation, and Thereby Attenuates GRP78-Modulated Oxidative Stress Balance and Cell Invasion

**Figure 5:**
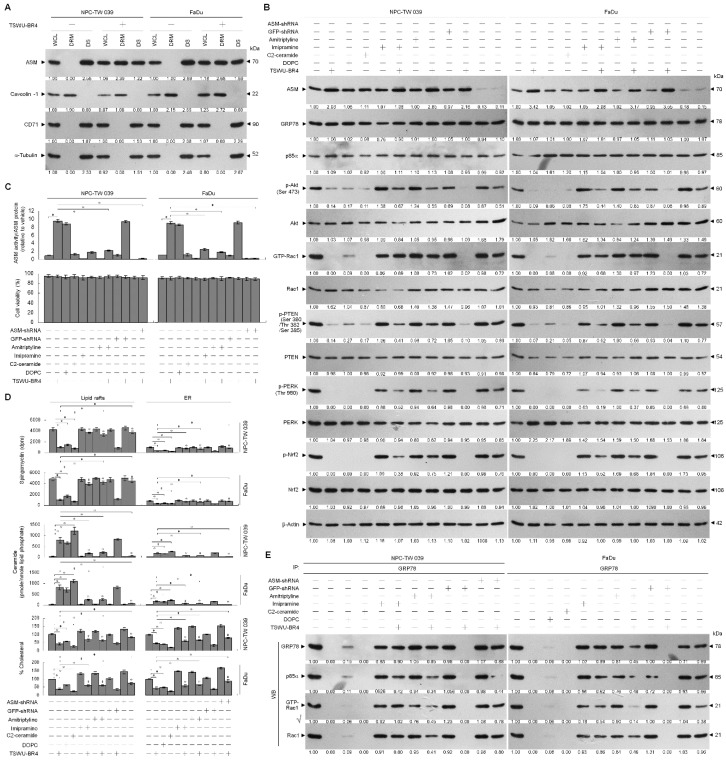

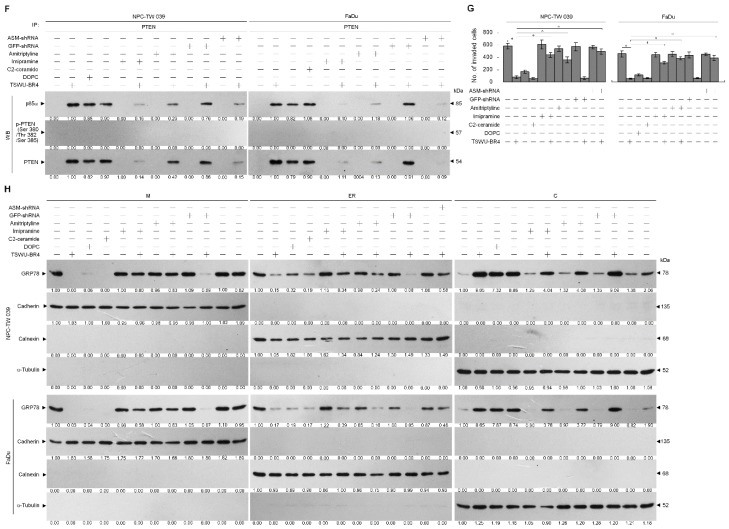
Lipid raft membrane-associated ASM activity is involved in TSWU-BR4-induced inhibition of GRP78-modulated oxidative stress balance and cell invasion. (**A**) Cells were treated with vehicle (−) or TSWU-BR4 (300 nM) for 36 h. DRM and DS fractions were prepared by flotation on a sucrose density gradient. The levels of the indicated proteins in the lysates of vehicle- or TSWU-BR4-treated DRM and DS fractions were determined by Western blot analysis using specific antibodies. At 12 h after transfection with an empty vector, GFP shRNA or ASM shRNA cells were treated with either vehicle (−), TSWU-BR4 (300 nM), DOPC (15 nmole), C2-ceramide (6 μM), imipramine (5 μM), TSWU-BR4 (300 nM) plus imipramine (5 μM), amitriptyline (8 μM), or TSWU-BR4 (300 nM) plus amitriptyline (8 μM) for 36 h. (**B**) The levels of the indicated proteins in the cell lysates were determined by Western blot analysis with specific antibodies. β-Actin was used as an internal control for sample loading. (**C**) ASM activities were determined by Acidic Sphingomyelinase Assay Kit. Cell viability was determined by the flow cytometric analysis of PI uptake. (**D**) The lipids were extracted from the DRM or ER fractions, and cholesterol, sphingomyelin, and ceramide were quantitated by Amplex Red Cholesterol Assay Kit and thin-layer chromatography, respectively. Ceramide concentrations were normalized to phospholipid phosphate. (**E**,**F**) The antibodies used for co-immunoprecipitation are indicated at the top of the figure. The proteins in the immunoprecipitated complexes from the DRM were analyzed by Western blot using specific antibodies. (**G**) The invaded cell numbers were assessed by the Matrigel invasion assays. (**H**) The levels of the indicated protein in the extracts of M, ER, and C fractions prepared from the cells treated as described above and determined by Western blot analysis using specific antibodies. The values are presented as the S.E.M. of three independent experiments. * *p* < 0.05: significantly different from empty vector-transfected TSWU-BR4-treated cells.

Because localization of acid sphingomyelinase (ASM) to the lipid raft membranes is an important factor for lipid raft recruitment and phosphatase activity on Akt of PTEN [37], we thought that lipid raft membrane localization of PTEN in the TSWU-BR4-treated cells might be caused by an action of ASM upon generation of ceramide within the lipid raft membranes. To verify this possibility, we treated cells with specific inducer, inhibitor, or shRNA for ASM and then performed a sucrose density-based membrane flotation to isolate the caveolin-1- and CD55-enriched DRM fractions or a subcellular fractionation to isolate the membrane (M), ER, and cytosol (C). The protein level and activity of ASM were increased after TSWU-BR4 treatment (Figure 5B,C). TSWU-BR4 also caused the induction of the lipid raft membrane-associated ASM, which was reduced in the DS level of ASM (Figure 5A). 1,2-dioleoyl-*sn*-glycero-3-phosphocholine (DOPC), a phosphatidylcholine, has the potential to induce activation of the ASM triggering of ceramide generation through sphingomyelin hydrolysis [38]. The treatment of cells with DOPC enhanced ASM activity, level of lipid raft and ER ceramide, and the lipid raft membrane-associated p85α–unphosphorylated PTEN complex formation and decreased levels of lipid raft and ER cholesterol, p-Akt (Ser 473), GTP-Rac1, p-PTEN (Ser 380/Thr 382/Ser 385), p-PERK (Thr 980), p-Nrf2, and cell invasiveness; this effect was similar to that of the treatment of TSWU-BR4 or C2-ceramide (Figure 5B,C,D,E,G). Consistent with the finding in TSWU-BR4-treated cells, treatment of DPOC or C2-ceramide in cells resulted in decreased levels of GRP78 in the plasma membrane and ER compared with vehicle-treated cells, whereas an increase of GRP78 level was observed in cytosol (Figure 5H). The co-treatment of ASM specific inhibitor (imipramine or amitriptyline) [39] along with TSWU-BR4 restored the cholesterol and sphingomyelin levels of lipid raft and ER, GRP78−p85α−GTP-Rac1 complex formation, levels of p-Akt, GTP-Rac1, p-PTEN (Ser 380/Thr 382/Ser 385), p-PERK (Thr 980), p-Nrf2, membrane/ER-associated GRP78, and invaded cell number (Figure 5B,C,D,E,G,H). Co-immunoprecipitation analyses of PTEN-associated complexes revealed a substantial reduction in the level of p85α–unphosphorylated PTEN complexes following imipramine or amitriptyline addition in TSWU-BR4-treated cells (Figure 5F). This impact was not from the induction of apoptotic cell death, because imipramine or amitriptyline does not show any significant reduction in cell viability (Figure 5C). Knockdown of ASM in cells did not affect the levels of p-Akt (Ser 473), p-PTEN (Ser 380/Thr 382/Ser 385), p-PERK (Thr 980), Nrf2; Rac1 activation; and cell viability (Figure 5B,C). Plasma membrane and ER association of GRP78 was not impaired in cells with knockout of ASM (Figure 5H). Cells expressing ASM shRNA in the presence of TSWU-BR4 exhibited rescue effects on the suppression the phosphorylation of Akt (Ser 473), PTEN (Ser 380/Thr 382/Ser 385), PERK (Thr 980) and Nrf2, Rac1 activation, GRP78−p85α−GTP-Rac1 complex formation, plasma membrane/ER association of GRP78, cholesterol, sphingomylein, and cell invasion, and as expected, p85α was not co-immunoprecipitated with PTEN (Figure 5B,C,D,E,F,G,H). These results indicate that TSWU-BR4 selectively acts on ASM to alter the lipid property of the lipid raft and ER membranes, which leads to alteration of GRP78 membrane trafficking and then disruption of GRP78−p85α−GTP-Rac1-mediated and ER-associated GRP78-mediated signaling from the lipid raft platform causing the oxidative stress-induced inhibition of cell invasion.

### 3.5. TSWU-BR4-Induced Ceramide Generation Attenuates Cell Invasion by Suppressing the PI3K–Akt-Regulated NF-κB-Mediated MMP-2 Expression Pathway

**Figure 6:**
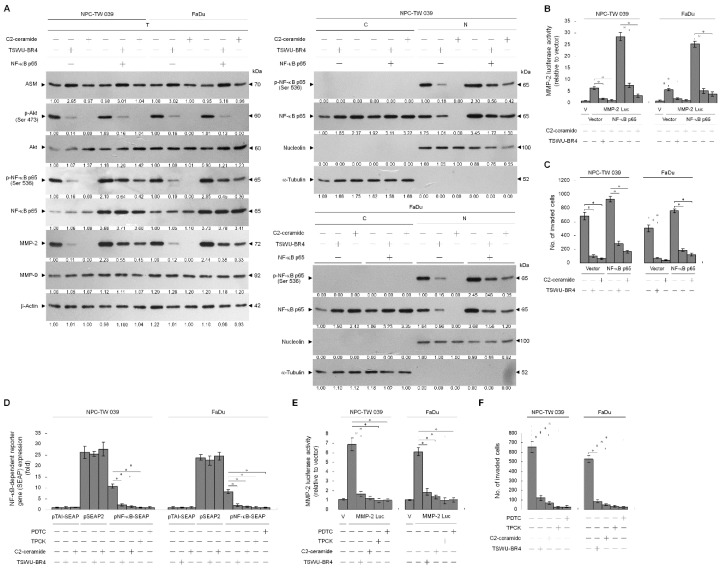
Ceramide generation is involved in TSWU-BR4-induced inhibition of GRP78−PI3K−Akt−NF-κB-mediated MMP-2 expression and cellular invasiveness. (**A**) At 12 h after transfection with the indicated plasmids, the cells were treated with the indicated compounds for an additional 36 h. The levels of the indicated proteins in the whole-cell, cytosolic, and nuclear extracts were then determined with specific antibodies. α-Tubulin and nucleolin were measured as internal controls for the cytosolic and nuclear fractions. The invaded cell numbers were assessed by Matrigel invasion assays. The values are presented as the S.E.M. of three independent experiments. * *p* < 0.05: significantly different from the vehicle-treated and vector-transfected cells or C2-ceramide-treated and vector-transfected cells. (**B**,**C**,**D**,**E**) At 12 h after co-transfection with the indicated reporter plasmids, the cells were treated with the indicated compounds for an additional 36 h. The SEAP, MMP-2 promoter, and invasive activities were then determined as described in Material and Methods. * *p* < 0.05: significantly different from the vehicle-treated, pNF-κB SEAP-transfected cells; the vehicle-treated, MMP-2 promoter-transfected cells; or the vehicle-treated cells.

PI3K–Akt signaling is reported to be involved in cancer cell invasion by modulating NF-κB-mediated MMP-2 expression through the control of Rac1 activity [35,40,41]. Hence, we next investigated the possible association of ASM-mediated PI3K–Akt signaling and NF-κB-dependent expression of MMP-2. NF-κB p65, MMP-2 promoter-driven luciferase reporter, and NF-κB-responsive secreted alkaline phosphatase (SEAP) reporter were ectopically expressed in TSWU-BR4-treated NPC cells and subsequently evaluated NF-κB-dependent SEAP activity, MMP-2 promoter activity, and cellular localization and phosphorylation of NF-κB. TSWU-BR4 treatment inhibited Akt (Ser 437) phosphorylation, NF-κB p65 (Ser 536) phosphorylation, p-NF-κB p65 nuclear translocation, NF-κB-dependent SEAP activity, MMP-2 promoter activity, and cell invasion in a manner similar to that occurring with C2-ceramide. An increased level and nuclear localization of p-NF-κB (Ser 536), MMP-2 expression, and cell invasive activity were observed in cells that overexpressed NF-κB. Ectopic expression of NF-κB resulted in resistance to TSWU-BR4- or C2-ceramide-induced abrogation of MMP-2 expression and cell invasion (Figure 6A,B). Treatment with TPCK or PDTC was shown to selectively inhibit NF-κB activity, resulting in significantly attenuated NF-κB-dependent SEAP activity, MMP-2 promoter activity, and cell invasive activity. This result is consistent with the finding in TSWU-BR4- or C2-ceramide-treated cells (Figure 6D,E,F). Thus, induction of ASM-mediated ceramide generation by TSWU-BR4 disrupted the lipid raft membrane-associated GRP78−p85α−GTP-Rac1-mediated signaling and then impaired NF-κB-mediated MMP-2, triggering the inhibition of NPC cell invasion.

## 4. Discussion

**Figure 7:**
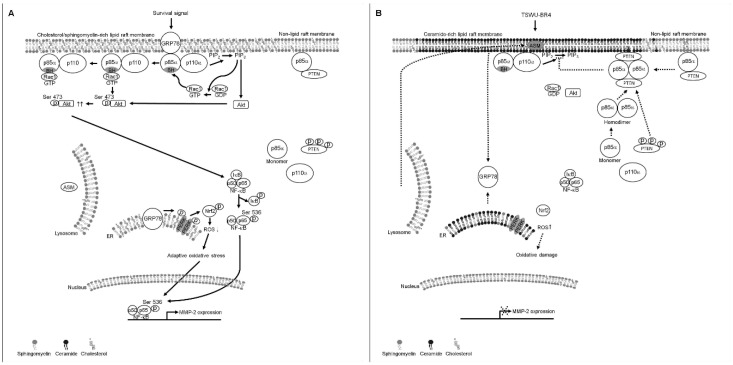
A molecular model for the induction of the inhibition of cancer cell invasion by TSWU-BR4. (**A**) The selective interaction of clustered GRP78−PI3K−Akt−GTP-Rac1 signaling molecules in the lipid raft membranes constitutes a central element in the initiation of ER lipid raft membrane-associated GRP78−PERK−Nrf2-mediated oxidative stress balance and NF-κB−MMP-2-mediated cell invasion in response to survival signals. (**B**) Under the condition of cellular TSWU-BR4 uptake, TSWU-BR4 triggers the lipid raft membrane translocalization of ASM from lysosome to promote the generation of ceramide-rich lipid raft membranes, thus inducting the formation of p85α–unphosphorylated PTEN tetrameric complexes and thereby disturbing the interaction between GRP78 and p85α in the lipid raft membranes. The lipid raft membrane targeting of ASM also lead to the translocation of GRP78 from the ER into the cytosol by displacing cholesterol with ceramide in the ER lipid raft membranes, causing oxidative damage-induced inhibition of cell invasion. The resulting lipid raft membrane-associated p85α–PTEN complexes can negatively regulate Akt activity and attenuate Rac1 activation by dephosphorylating the 3-position of PIP_3_ to PIP_2_. Akt inactivation was accompanied by attenuation of NF-κB-mediated MMP-2 expression, which can result in the suppression of the invasive activity of cancer cells.

Unlike the plasma membrane, lipid raft membranes are enriched in a high cholesterol and sphingolipid content and packed in a liquid-order structure. This distinctive property allows recruitment of specific receptors and signal molecules to form a signaling platform for biosynthetic pathway in cancer cells [2,42]. Therapeutic targeting or disrupting of lipid raft membrane-associated signal molecules or alteration in the cholesterol content of lipid raft membranes has been considered as a potential anticancer strategy [43,44]. Our findings indicate that bichalcone analog TSWU-BR4-induced translocalization ability of ASM to the lipid raft membranes was showed for targeting PTEN to interact with p85α at the lipid raft membranes and stimulating PTEN-mediated inactivation of Akt and Rac1 by depleting cholesterol from the lipid raft membranes; it also caused dissociation of GRP78 with p85α and delocalization of lipid raft and ER membrane GRP78 into the cytosol, which was, however, overcome by treatment of imipramine, amitriptyline treatment, or ASM shRNA. The restoration of the formation of lipid raft membrane-associated GRP78−p85α−GTP-Rac1 complexes, ROS and cholesterol levels, and cell invasive activity and suppression of p85α−PTEN complex formation were observed in the presence of ectopic expression of GRP78 with TSWU-BR4 treatment. Forced expression of GRP78 in cells increased the localization of GRP78 to the lipid raft membrane, which enhanced levels of p-Akt (Ser 473), p-PTEN (Ser 380/Thr 382/Ser 385), p-PERK (Thr 980), p-Nrf2, Rac1-GTP, cellular cholesterol, and invaded cell number, as well as decreased ROS generation. Membrane localization of GRP78 was implicated in playing a positive regulator of the oncogenic signaling cascade [45]. The physiological relevance of cell surface GRP78 in cancer cells is thought to act as a signal activator that binds to the p85α regulatory subunit of PI3K for initiating the PI3K−Akt signaling pathway to resist apoptosis in response to ER stress [13]. Beside the role of GRP78 in regulating the protein homeostasis of ER, it controls the cholesterol metabolism and biosynthesis of membrane lipids by modulating gene expression involved in oxidative stress signaling to maintain low levels of ROS [46]. Indeed, ER membrane-associated GRP78 activated PERK−Nrf2 signaling under stress condition, which was advantageous for maintaining ROS homeostasis to prevent cell damage and promote cancer cell survival [19,47]. Elevated cholesterol in the lipid raft membrane has been implicated in stimulating phosphorylation of Akt (Ser 473) and oncogenic activity of PI3K in cancer cells [48], whereas blocking the cholesterol accumulation impaired cancer cell invasion [49]. Consistent with the finding in TSWU-BR4 treatment, lipid raft membrane targeting the interaction of PTEN with p85α by C2-ceramide treatment attenuates PI3K−Akt−Rac1 signaling by disrupting the interaction between GRP78 and p85α and causes translocation of GRP78 from the lipid raft membrane and ER into the cytosol. C2-ceramide not only selectively depleted lipid raft cholesterol but also decreased the cholesterol content of ER cholesterol. The presence of ceramide by ASM-mediated sphingomyelin hydrolysis in lipid raft is sufficient to displace cholesterol from the lipid rafts [37,50]. Moreover, it is well documented that cholesterol of lipid rafts can be selectively displaced by ceramide treatment [11]. The finding showed that pancreatic cells with decreased sphingomyelin content in ER lead to disruption of ER lipid rafts triggering ER stress-induced deregulation of lipid metabolism [51]. A link to exert enzymatic activity of PTEN as a PIP3 phosphatase has been described by formatting a p85α−PTEN complexes in the plasma membrane [7,52]; however, we observed only a small amount of p85α−unphosphorylated PTEN complexes in the plasma membrane of vehicle-treated cancer cells, which in turn changes and increases localization in the lipid rafts membrane when exposed to TSWU-BR4. Other work has suggested that the lipid phosphatase activity and membrane association of PTEN is enhanced by modulating the formation of p110-free homodimerized p85α–unphosphorylated PTEN tetrameric complexes in the plasma membrane [9]. Collectively, we propose a working model whereby by TSWU-BR4 can induce inhibition of cancer cell invasion (Figure 7): TSWU-BR4 induction of the formation of ceramide-rich lipid raft membrane-associated p110-free homodimerized p85α–unphosphorylated PTEN tetrameric complexes by triggering the lipid raft membrane translocation of ASM to catalyze the hydrolysis of sphingomyelin to ceramide. The resultant ceramide displaces cholesterol from the lipid raft and ER membranes to disrupt the interaction between GRP78 and p85α and cause disassociation of GRP78 with the lipid raft and ER membranes, thereby attenuating the regulatory effects of GRP78 on the control of oxidative stress balance, cholesterol metabolism, and PI3K−Akt−Rac1−NF-κB signal pathway. We also provide new evidence that intact lipid raft membranes appear to be required for the generation, maintenance, and functionality of GRP78−PI3K−Akt−GTP-Rac1 effector molecules. The existence and role of lipid raft membrane-associated GRP78−PI3K−Akt−GTP-Rac1 molecules are providing new insights into the dynamic process occurring in the membrane of cancer cells.

In most types of human normal cells, p85α is predominantly present in a homo-dimeric form because of the relatively low amount of p110α, and it acts as a negative regulator of PI3K−Akt signal pathway to control cell proliferation and survival [9,31,53,54,55,56]. Studies reported by Zhang et al. have shown that human non-tumor nasopharyngeal epithelial tissues exhibit a greater PTEN level than NPC tissues. GRP78 was found to be expressed highly and localized preferentially on the cell surface of a variety of human cancer types, whereas its expression and localization are not on the cell surface of normal human cells [57]. Both phosphorylated p85α (Thy 508) and Akt (Ser 473) were observed in the lipid rafts of normal human embryonic lung WI-38 fibroblasts, whereas no p85α−Rac1 complexes and GTP-Rac1 were detected in this cells [35]. We propose that the potential lack of an inhibitory effect of TSWU-BR4 on human normal cells, which might be correlated with a high amount of PTEN and the absence of cell surface GRP78, is responsible for the formation of p110-free homodimerized p85α–unphosphorylated PTEN tetrameric complexes in the lipid raft membranes, resulting in PTEN activation through regulation of Akt activity by dephosphorylating 3-position of PIP_3_ to PIP_2_ in normal cells. Therefore, TSWU-BR4-triggered lipid raft membrane association of ASM may not be effective in inducing inhibition of normal cell proliferation.

In summary, the study highlights that specific induction in the lipid raft membrane localization of ASM contributes attenuation of the oncogenic functions of membrane-associated GRP78 in the modulation of oxidative stress, cholesterol metabolism, and cell invasion during exposure of TSWU-BR4. This finding not only has potential implications for understanding the molecular mechanism of TSWU-BR4 inhibition of cancer cell invasion but also provides a theoretical basis for the further development of novel lipid raft membrane-associated signal molecule inhibitors.

## 5. Conclusions

This is the first finding that TSWU-BR4-triggered ceramide-enrich lipid raft membrane platform formation to initiate formation of p85α–unphosphorylated PTEN complexes by inducing lipid raft membrane targeting of ASM, leading to the dislocalization of GRP78 from the lipid raft and ER membranes to the cytosol and suppression of the interactions between p85α and GRP78, thereby attenuating the GRP78-regulated oxidative stress balance and cholesterol metabolism and the GRP78−p85α−Akt−GTP-Rac1−NF-κB−MMP-2-mediated cancer cell invasion. Therefore, the molecular mechanisms underlying TSWU-BR4-induced inhibition of cancer cell invasion may reveal strategy for theoretical basis of new concepts in anticancer treatment.

## Supplementary Materials

The following are available online at https://www.mdpi.com/2073-4409/9/2/371/s1, Figure S1: The effect of TSWU-BR4 on the cell surface localization of GRP78.

## Abbreviations

Acid sphingomyelinase	ASM
Endoplasmic reticulum	ER
Glucose regulated protein 78	GRP78
Matrix metalloproteinase-2	MMP-2
Nasopharyngeal carcinoma	NPC
Nuclear factor erythroid-2-related factor 2	Nrf2
Nuclear factor-kappa B	NF-κB
Phosphatase and tensin homolog deleted from chromosome 10	PTEN
Phosphatidylinositol 3-kinase	PI3K
Phosphatidylinositol-4,5-bisphosphate	PIP_2_
Phosphatidylinositol-3,4,5-trisphosphate	PIP_3_
Protein kinase B	Akt
Protein kinase RNA-like endoplasmic reticulum kinase	PERK
Ras-related C3 botulinum toxin substrate 1	Rac1
Reactive oxygen species	ROS
Short hairpin RNA	shRNA
Human pharyngeal squamous carcinoma	PSC
